# Electronic data collection for multi-country, hospital-based, clinical observation of maternal and newborn care: EN-BIRTH study experiences

**DOI:** 10.1186/s12884-020-03426-5

**Published:** 2021-03-26

**Authors:** Harriet Ruysen, Ahmed Ehsanur Rahman, Vladimir Sergeevich Gordeev, Tanvir Hossain, Omkar Basnet, Kizito Shirima, Qazi Sadeq-ur Rahman, Sojib Bin Zaman, Nisha Rana, Nahya Salim, Tazeen Tahsina, Georgia R. Gore-Langton, Shafiqul Ameen, Dorothy Boggs, Stefanie Kong, Louise T. Day, Shams El Arifeen, Joy E. Lawn, EN-BIRTH Study Group, Qazi Sadeq-ur Rahman, Ahmed Ehsanur Rahman, Tazeen Tahsina, Sojib Bin Zaman, Shafiqul Ameen, Tanvir Hossain, Abu Bakkar Siddique, Aniqa Tasnim Hossain, Tapas Mazumder, Jasmin Khan, Taqbir Us Samad Talha, Rajib Haider, Md. Hafizur Rahman, Anisuddin Ahmed, Shams Arifeen, Omkar Basnet, Avinash K. Sunny, Nishant Thakur, Regina Gurung, Anjani Kumar Jha, Bijay Jha, Ram Chandra Bastola, Rajendra Paudel, Asmita Paudel, K. C. Ashish, Nahya Salim, Donat Shamba, Josephine Shabani, Kizito Shirima, Menna Narcis Tarimo, Godfrey Mbaruku, Honorati Masanja, Louise T. Day, Harriet Ruysen, Kimberly Peven, Vladimir S. Gordeev, Georgia R. Gore-Langton, Dorothy Boggs, Stefanie Kong, Angela Baschieri, Simon Cousens, Joy E. Lawn

**Affiliations:** 1 Maternal, Adolescent, Reproductive & Child Health (MARCH) Centre, London School of Hygiene & Tropical Medicine, London, UK; 2 Maternal and Child Health Division, International Centre for Diarrhoeal Disease Research, Bangladesh (icddr,b), Dhaka, Bangladesh; 3 Institute of Population Health Sciences, Queen Mary University of London, London, UK; 4 Research Division, Golden Community, Lalitpur, Nepal; 5 Department of Health Systems, Impact Evaluation and Policy, Ifakara Health Institute, Dar Es Salaam, Tanzania; 6 Department of Paediatrics and Child Health, Muhimbili University of Health and Allied Sciences, Dar Es Salaam, Tanzania

**Keywords:** Data management, Software, Electronic data collection tools, Electronic health records, Hospital records, Maternal, Newborn, Birth, Observation

## Abstract

**Background:**

Observation of care at birth is challenging with multiple, rapid and potentially concurrent events occurring for mother, newborn and placenta. Design of electronic data (E-data) collection needs to account for these challenges. The *Every Newborn* Birth Indicators Research Tracking in Hospitals (EN-BIRTH) was an observational study to assess measurement of indicators for priority maternal and newborn interventions and took place in five hospitals in Bangladesh, Nepal and Tanzania (July 2017–July 2018). E-data tools were required to capture individually-linked, timed observation of care, data extraction from hospital register-records or case-notes, and exit-survey data from women.

**Methods:**

To evaluate this process for EN-BIRTH, we employed a framework organised around five steps for E-data design, data collection and implementation. Using this framework, a mixed methods evaluation synthesised evidence from study documentation, standard operating procedures, stakeholder meetings and design workshops. We undertook focus group discussions with EN-BIRTH researchers to explore experiences from the three different country teams (November–December 2019). Results were organised according to the five a priori steps.

**Results:**

In accordance with the five-step framework, we found: 1) Selection of data collection approach and software: user-centred design principles were applied to meet the challenges for observation of rapid, concurrent events around the time of birth with time-stamping. 2) Design of data collection tools and programming: required extensive pilot testing of tools to be user-focused and to include in-built error messages and data quality alerts. 3) Recruitment and training of data collectors: standardised with an interactive training package including pre/post-course assessment. 4) Data collection, quality assurance, and management: real-time quality assessments with a tracking dashboard and double observation/data extraction for a 5% case subset, were incorporated as part of quality assurance. Internet-based synchronisation during data collection posed intermittent challenges. 5) Data management, cleaning and analysis: E-data collection was perceived to improve data quality and reduce time cleaning.

**Conclusions:**

The E-Data system, custom-built for EN-BIRTH, was valued by the site teams, particularly for time-stamped clinical observation of complex multiple simultaneous events at birth, without which the study objectives could not have been met. However before selection of a custom-built E-data tool, the development time, higher training and IT support needs, and connectivity challenges need to be considered against the proposed study or programme’s purpose, and currently available E-data tool options.

**Supplementary Information:**

The online version contains supplementary material available at 10.1186/s12884-020-03426-5.

## Key findings

**Table:** 

**What was known before?** • Implementation and use of electronic data (E-data) capture are increasing worldwide. Few published papers have examined the process and learning from large, multi-site observational data collection, especially for facility-based intrapartum care. Design choices may vary according to the purposes, data type, local context, capacity and number of data collectors. **What was done?** • We applied a five-step framework to evaluate EN-BIRTH study processes including design and use of a custom-built E-data capture system in five hospitals, in three low- and middle-income countries (LMICs), with variable internet connectivity. For this article, we undertook descriptive analyses of relevant study documentation (protocols, operating procedures etc.) and focus group discussions exploring the research team’s experience regarding design and implementation of E-data collection. These findings have implications for E-data development and use in other LMIC settings during research/surveys or programme monitoring. **What did we learn from each step?** • **Step 1) Selection of EN-BIRTH study data collection approach and software** E-data capture platforms vary in complexity, adaptability and cost. A systematic selection process is helpful based on purpose, and non-negotiable characteristics in order to achieve the study objectives. EN-BIRTH needed to collect time-stamped clinical observation data for > 23,000 women and newborns in labour wards, operation theatres, and kangaroo mother care wards. Exit-survey interviews were conducted, and register-record and case-note data were extracted. Hence a custom-built system was required since there was no suitable E-data data capture tool available on the market. • **Step 2) Design of data collection tools and programming** The transition from paper to app-based tools required in-depth consultation with data collectors, various tool users, and piloting, involving an iterative process that took more time than anticipated. Finalising variable lists and permitted data ranges early during software development process were fundamental. • **Step 3) Recruitment and training of data collectors** Standardised training materials were essential with skills-based sessions focused on the study objectives, research procedures, and competency-based use of the software are key. • **Step 4) Data collection, quality assurance, and improvement** A collaborative, multi-directional learning network of South-South and also North-South learning was valued and helped by regular, multisite virtual calls, sharing progress by site based on the data monitoring dashboard, and also sharing local solutions with other teams for peer-to-peer learning. Inclusion of facility-level stakeholders in the planning and organisation of data collection was essential to avoid disruptions to routine services. • **Step 5) Data management, cleaning and analysis** E-data collection was perceived to reduce data cleaning challenges and to reduce erroneous entries however, open text fields and data captured in four different languages requiring back translation were still time consuming during analyses. **What next?** • Our custom-built E-data tool had advantages including the user-friendly interface, time-stamping, increased data security, real-time monitoring, and inbuilt data quality measures. However, careful assessment of the context and people-time costs are needed and custom-built software should only be considered if existing E-data platforms are not able to meet the objectives of a given research or health programme.	

## Background

Around 80% of births worldwide are estimated to occur in facilities [1], however the large increase in institutional births has not led to the expected reductions for maternal and newborn mortality in low- and middle-income countries (LMICs) [1–4]. This quality gap has led to multiple studies to assess the content and experience of care during labour and birth [5–11], and a new focus on the validity of recall surveys and routine measurement [12–16]. However, given the potential for concurrent events and delivery of multiple health interventions during labour and birth, real-time observation of intrapartum care is complex. Several validation studies have included the use of paper-based intrapartum observation checklists [12–16]. Observer checklists have been implemented using smartphones and tablets in a large study observing intrapartum care in six countries in Africa [17, 18], and in one Tanzanian study where 1049 babies were observed during birth and the early postpartum period [10]. However, there is little information about software selection and no published data exploring these experiences.

E-data capture is increasingly utilised within both programmes and research, and is usually implemented via mobile devices such as smartphones and tablets. E-data collection can be time-saving with direct data capture minimising time spent digitalising paper-based forms, and pre-programmed skip patterns increasing data collector’s efficiency and data quality [19–21]. Such E-data features have also been shown to reduce erroneous data entry [22, 23]. Consequently, E-data capture is now the primary approach for both the Demographic and Health Surveys Program (DHS) and the Multiple Indicator Cluster Surveys (MICS)—nationally representative household surveys providing critical health information in more than 90 countries [24, 25]. While there is increasing evidence evaluating survey-based E-data collection tools [19, 22, 26–30], there is little assessing E-data collection platforms for other types of data collection such as facility-based observation, or register-record extraction [21, 23].

The *Every Newborn–* Birth Indicators Research Tracking in Hospitals (EN-BIRTH) study, was an observational study of > 23,000 hospital births in three LMICs (Tanzania, Bangladesh and Nepal). EN-BIRTH focused on validation of indicators prioritised within the *Every Newborn* measurement improvement roadmap (uterotonics for prevention of post-partum haemorrhage, early initiation of breastfeeding, neonatal resuscitation, kangaroo mother care (KMC), antenatal corticosteroids and inpatient management of neonatal infections) [31, 32]. EN-BIRTH study included five comprehensive emergency obstetric and neonatal care (CEmONC) hospitals (Additional file 1). Clinical observations were continuous during labour, birth, and the immediate postpartum period on the labour and delivery wards, and intermittent on the KMC wards. Exit-survey interviews were conducted, and register-record data extraction was undertaken in five sites. Observation was not feasible for inpatient care of newborn infections or administration of antenatal corticosteroids, so for these cases, data-extraction from clinical records/case notes was also used. All sites were subject to variable internet connectivity and power disruptions. Detailed methods, as well as the overall validity results, are reported separately [31, 33].

**Fig. 1:**
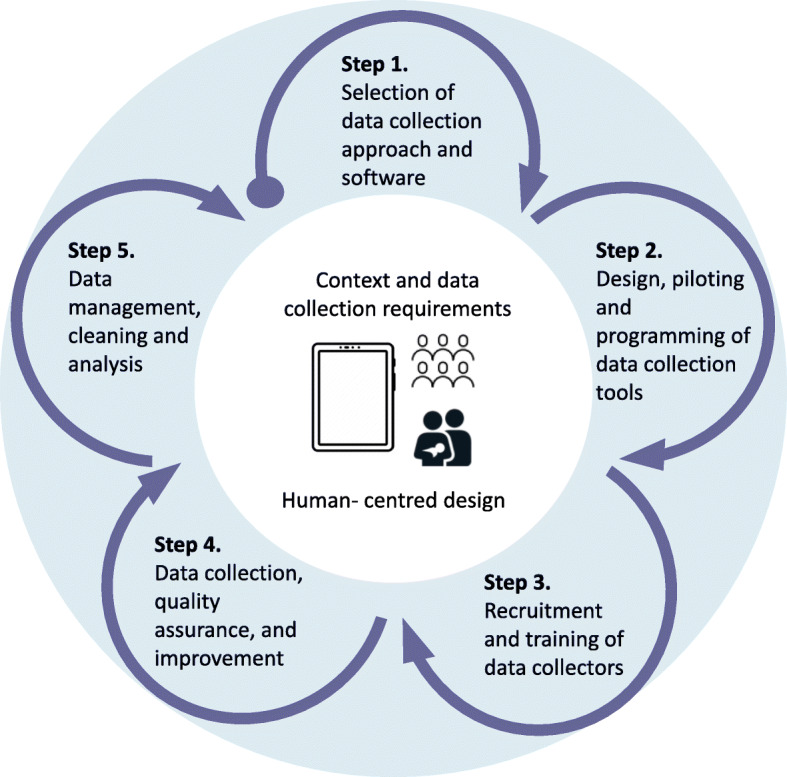
Five-step framework for data collection and implementation of a large multi-country observational study

A linked study, EN-INDEPTH, was undertaken in parallel and focused on data collection in population-level surveys to improve measurement of pregnancy outcomes [34]. Recognising a similar systematic approach was required in both studies to design data collection systems, especially for E-data tools, a five-step framework was jointly developed between the two research teams [30] (Fig. 1). Using human-centred design principles, we describe and apply the same five steps to synthesise learning from these two processes with implications for other research studies or programmes (Fig. 1). Given differences in purpose of the two studies, and differing challenges, the eventual choice of tools and processes differed and enable common learning regarding the various steps, considering users’ reality, experiences and needs [35].

## Objectives

This paper is part of a supplement based on the EN-BIRTH multi-country validation study, *‘Informing measurement of coverage and quality of maternal and newborn care’*. This paper is organised by the five steps identified for the E-data tool design, and implementation process (Fig. 1). We undertook a mixed methods evaluation as follows:

- 1. **To synthesise the process for designing EN-BIRTH study DATA COLLECTION METHODS** using study documentation in accordance with the five steps, with synthesis of learning per step.
- 2. **To explore qualitative data on the experiences of EN-BIRTH data managers** and study implementers according to the five steps.

## Methods

We employed mixed methods to document the development, use, and users’ perspectives on the tool, guided by the five-step conceptual framework (Fig. 1).

### Study setting

EN-BIRTH study included five comprehensive emergency obstetric and neonatal care (CEmONC) hospitals: Maternal and Child Health Training Institute, Azimpur and Kushtia General Hospital in Bangladesh, Pokhara Academy Health Sciences in Nepal, and Muhimbili National Hospital and Temeke District Hospital in Tanzania. EN-BIRTH study participants were consenting women admitted to the labour and birth wards in the five study hospitals. Data collection was undertaken between July 2017 and July 2018 (Additional file 1). Observers worked in shifts to provide 24 h observation and would hand over ongoing observations to the in-coming staff if necessary.

### Process evaluation

Our description of process is based on study documentation including standard operating procedures and protocols, workshop and meeting and minutes, email correspondence, and stakeholder reports. These inputs were synthesised to provide a process description in accordance with the five-step conceptual framework (Fig. 1).

### Focus group participants

A purposive sample of twelve participants was selected, eight were interviewed. The sample included three EN-BIRTH data managers, one co-principal investigator, and four study implementers who were involved in data analysis. Two of the participants also worked on the E-data tool software development. The sample included representation from each country research team: four from Bangladesh, and two from Tanzania and Nepal respectively. A further four participants were invited, but it was not possible to find a suitable meeting time. In addition, informal feedback was elicited with co-principal investigators at the London School of Hygiene & Tropical Medicine (LSHTM). As the data collectors were no longer employed by the study, they could not be included in the sample frame.

### Focus group methods

Focus group discussions (FGDs) were conducted during November and December 2019, using a structured guide to facilitate a dynamic discussion with opportunities to explore differences and similarities between site teams across all five development steps. We anticipated this was integral to identification of emerging themes.

Discussions took place via web-based conference calls and were in English with two LSHTM researchers present. The FGD guide (Additional file 2) was developed by project managers and the LSHTM team, and structured by the five-step framework (Fig. 1). This was aligned with the FGD guides used by EN-INDEPTH study [30]. Content was coded using NVivo (version 12) software. Emerging themes were included during the analysis and were coded as sub-categories within each step.

Interviews were audio recorded, transcribed and coded. Data were anonymised. The research team was small, so to protect participant confidentiality anonymization and analysis was undertaken by one researcher (HR), and checked with a second researcher (SK) not closely affiliated with study data collection. Anonymised data are stored on a secure password protected server only accessible by these two researchers.

To assess confirmability, credibility and dependability of the analysis, transcripts were shared with participants to be corrected where necessary. The preliminary and end-stage findings were also reviewed and discussed with participants and the senior authors. In addition, the overall findings and this manuscript were shared with the whole EN-BIRTH team who were asked to provide corrections, additional insights on the learnings, and implications.

Results were reported in accordance with the consolidated criteria for reporting qualitative research (COREQ) checklist (Additional file 3). We did not expand sampling beyond participants from the three country research teams, so it is difficult to assess if data saturation was reached. Ethical approval was granted by institutional review boards in all implementing countries and the London School of Hygiene & Tropical Medicine (Additional file 4).

## Results

Our results—process description and findings from the FGDs—are summarised according to the five-step framework (Fig. 1) as follows:

### Step 1: Selection of data collection approach and software

The study formative phase and data flow assessments (Additional file 5) highlighted characteristics necessary for a data collection tool to enable this complex data collection, observing simultaneous, rapid maternal and newborn events and health interventions in real-time. It was quickly apparent that paper-based observation checklists would be too complex, especially at the time of birth with multiple events happening quickly for the woman and baby, with researchers having to flip between long paper-based tools whilst following manual skip-patterns. EN-BIRTH labour ward observation checklists included multiple events that were not necessarily sequential and could coincide [36].

Based on the formative phase, the following requirements were identified for an E-data system:

- Participant flow management capacity (individual participant tracking, assignment allocation, observation reassignment, and linking the same woman to exit survey data entry, and register-record extraction).
- 24 h observation.
- Screen that allowed several processes and events to be recorded at once with rapid clicks (e.g. skin to skin initiation and administration of a uterotonic).
- Time-stamping of multiple variables.
- Access and use in accordance with five cadres of data collector (trackers, clinical observers, data extractors/verification officers, and supervisors or super-users).
- Pause function during observation, in case of adverse clinical events without appropriate health worker response where the observer may have to suspend an observation.
- Real-time data synchronisation to server, yet with offline data collection capability.
- Data security.

**Fig. 2:**
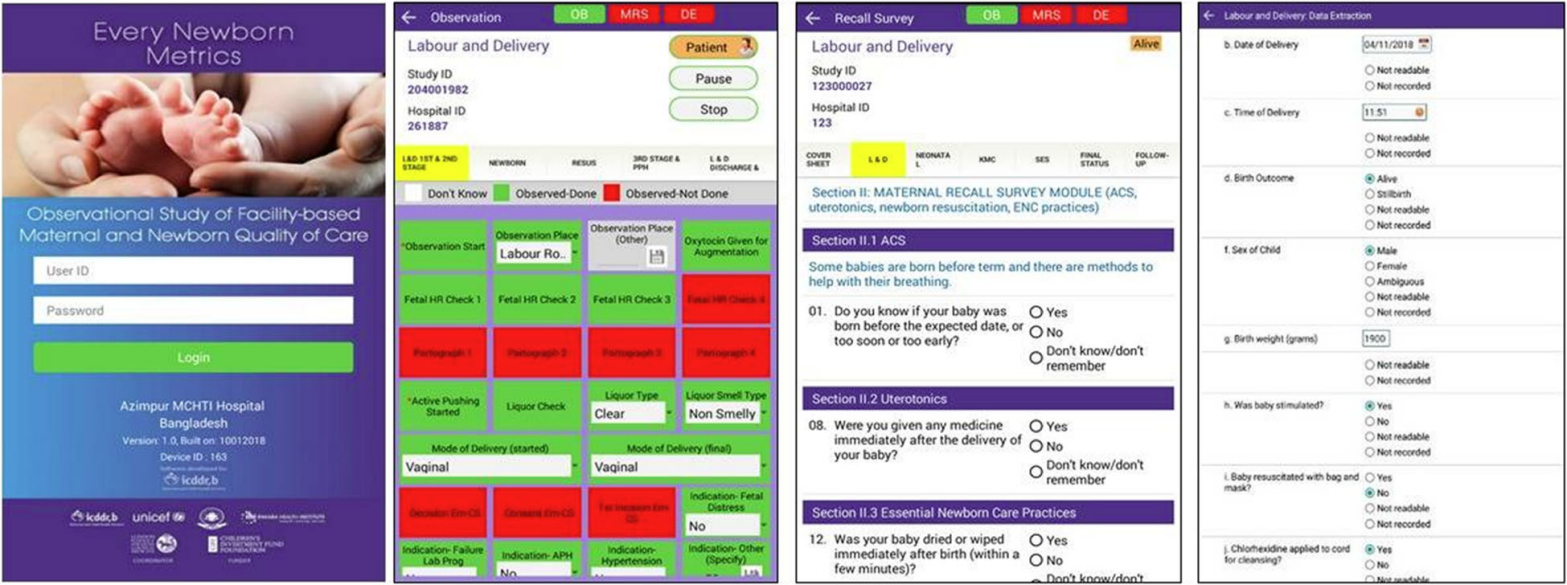
Android tablet-based data capture application, EN-BIRTH study

The research team had experiences with various software packages, such as REDCap, KoBo Toolbox, and Open Kit Data [37, 38]. These software packages were assessed against EN-BIRTH study requirements. None of these or other existing free and readily-available software met all the agreed requirements (Additional file 6); the EN-BIRTH team therefore elected to develop a custom-built E-data capture tool. The Bangladesh study team, led by International Centre for Diarrhoeal Disease Research, Bangladesh (icddr,b) had in-house software design capacity and experience of developing customised applications (apps) for large scale survey-based data collection, and therefore lead EN-BIRTH software development. The E-data system structure was agreed during a workshop (Tanzania, December 2016), and programmed by icddr,b in partnership with LSHTM and the Tanzanian and Nepalese research groups (Additional file 1). The app development team included expertise in information technology programming, data collection and management, statistical analysis, epidemiology, observational research and maternal and newborn health. Multidisciplinary perspectives are essential in bringing together diverse perspectives and experiences via a cooperative design process to innovate and reframe challenges from multiple perspectives [35]. The E-data tool had a multi-functional interface, colour coded command buttons, a range of checkboxes, radio buttons, drop-down lists, and pause and stop functionality (Fig. 2, Additional file 7).

All the EN-BIRTH teams had some previous experience using Android OS-operated tablets. Specifications for the tablets were agreed in accordance with the software needs, noting that a larger screen was deemed necessary to accommodate as many variables as possible on one screen for labour ward observation (Additional file 8).

#### Respondents’ perspectives on data collection approach and software

Respondents consistently cited E-data capture as advantageous for clinical observation, and reported that the proposed E-data app interface was extremely user-friendly:

*“…you could have 10 or 20 questions in a single stream and just press the button. It was really ideal for the kind of study we were doing where there was no systematic order for things to happen. It was almost impossible to do with a questionnaire because you would be flipping the page to turn over to one question and back from another.”*

-Researcher, Tanzania

### Step 2: Design, piloting and programming of data collection tools

EN-BIRTH included four different types of E-data collection tool (Fig. 2):

- Observation checklists for labour and KMC wards.
- Register-record extraction.
- Exit-interview surveys with women.
- Case-note extraction verification tool for newborn and antenatal records.

The EN-BIRTH E-data app allowed for different user roles with varying levels of permission and functionality: data collector (data collection), tracker (assigning and monitoring data collection by data collectors), supervisor (quality assurance audits), and the ‘super-user’ (E-data team, data management).

Design of the data collection tools was a multi-step process including review of relevant literature and stakeholder consultation. Observation checklists were collated from research studies [13, 14, 16, 39], the Maternal and Child Health Integrated Programme (MCHIP) [40], and the World Health Organization’s (WHO) Safe Childbirth Checklist [41]. These tools were expanded to include the numerator and denominators for the selected indicators to be validated in the EN-BIRTH study, with priority markers of quality of care as detailed in the published protocol [31]. The exit-survey forms were designed to capture woman’s report for all the variables required for validation, using existing questions in Demographic and Health Surveys (version 7) and/or Multiple Indicator Cluster Survey Questionnaire (version 5), or if needed new questions for those items not included before [42, 43]. The register data extraction forms also included all prioritised indicators [44]. Data collection tools were standardised against current WHO clinical guidelines for the provision of antenatal corticosteroids, prevention of post-partum haemorrhage, neonatal resuscitation, essential newborn care, KMC and treatment of inpatient neonatal infections [45–49]. Paper-based data collection tools were pilot-tested in late 2016 and transferred to the E-data app in early 2017 (Additional file 1).

Data collection tools were formatted into a variable matrix which was the basis for the final analysis code book. This was used to programme the E-data platform with active patient/respondent tracking system, and was adapted in accordance with health facility and data flow assessment results. The E-data app was translated into local languages for use in Bangladesh, Nepal, and Tanzania.

Hospital visits were undertaken for server set-up and to configure the database. All server infrastructure was checked for security and safety (appropriate software and hardware). Steps for regular server and tablet maintenance were agreed between all sites and included several updates, inspection for hardware errors, and regular secure data back-up (Additional file 9).

**Fig. 3:**
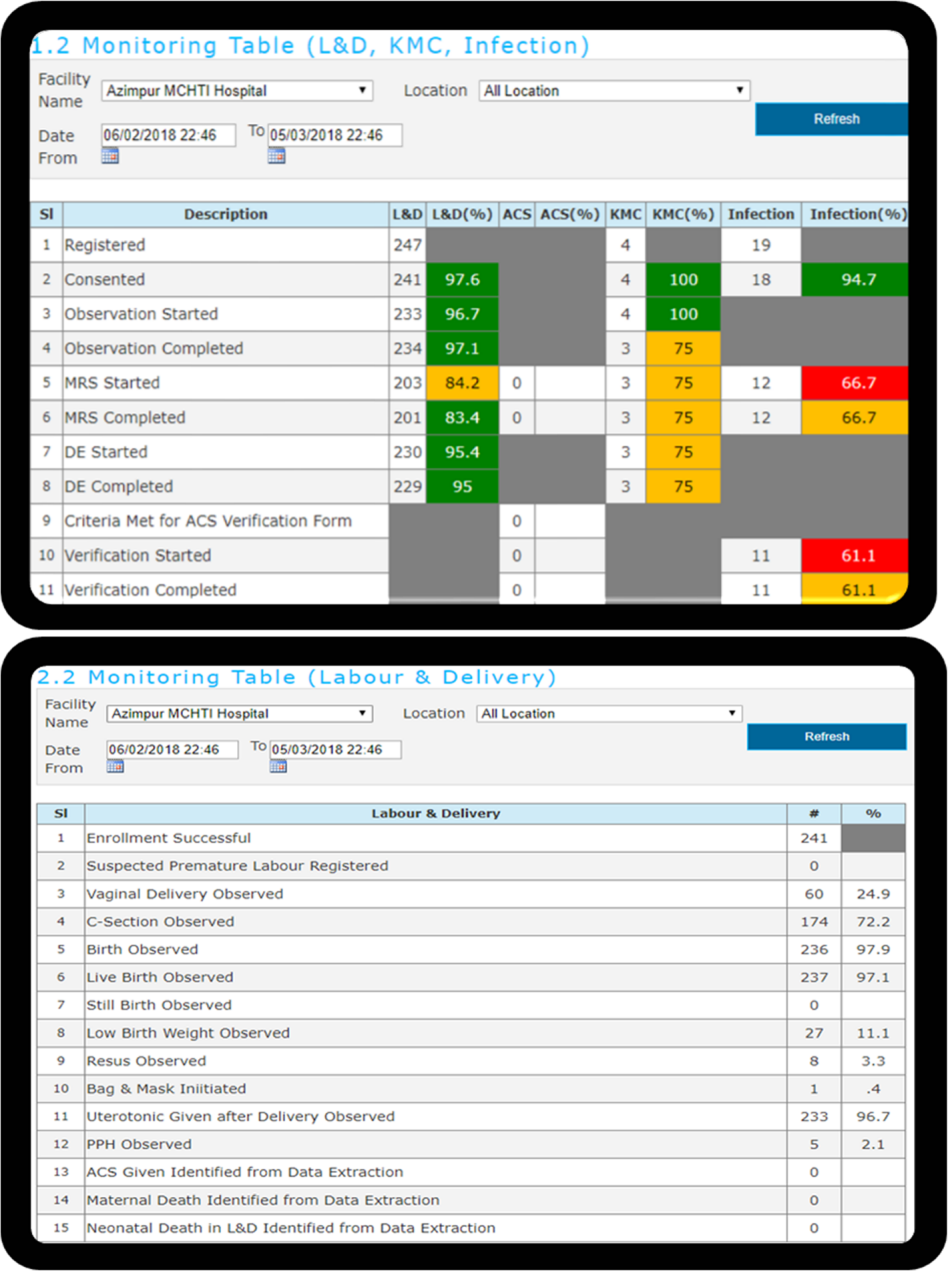
Data dashboard monitoring, EN-BIRTH study

Pilot testing was undertaken in phases and was fundamental to ensuring a user-focused design process that was iterative, and able to respond to user feedback [50]. This included fortnightly research team meetings throughout the E-data tool development process using test versions of the app, and finally 2 months of live testing ahead of data collector training. Programming of the custom-built tool was extremely complex and time consuming, requiring high levels of expertise and multiple rounds of pilot testing. The app was finalised with the addition of the data quality dashboard shortly after data collection commenced. The dashboard provided a linked overview of registered participants from consent to discharge tracking core study indicators and a data capture cascade for participants and completion of forms (Fig. 3).

#### Respondents’ perspectives on design and programming data collection tools

The observation interface of the E-data app was highly regarded by all participants who reported that it was essential to ensure accurate observation data within this study context:

*“We developed our own [app interface] to fulfil specific requirements: observation, time tracking, patient tracking, data monitoring of data collection.”*

-Researcher, Bangladesh

The EN-BIRTH study was a collaboration among teams across three implementing countries and LSHTM, with integral mechanisms to strengthen the multi-country networks and South-South sharing. This was facilitated via regular team calls, several workshops, and devolution of responsibility for specific outputs to smaller groups with representation from all four counties within the team. A designated website with secure file-sharing was also developed and maintained with current versions of country-specific E-data app installation files, as well as related documentation and user guides. Multi-site bi-weekly data management calls provided a platform for proactive trouble shooting, data management and ongoing review of operating procedures and progress, and were perceived as “*very helpful”*. This partnership approach was positively regarded by all respondents and created opportunities for learning and development:

*“We like the South – South collaboration.”*

-Researcher, Nepal

*“This was a unique thing for this project so for me was a positive thing compared to others.”*

-Researcher, Tanzania

However, coding of the EN-BIRTH E-data app was led by icddr,b and required a more centralised approach than other parts of the development process. This was contentious and other country team members expressed their frustration:

*“The country teams couldn’t really see or feel part of the app software development process.”*

-Researcher, Tanzania

*“We [assumed we] would build the capacity within our own teams on the app development process and other such things, but so much of it was controlled by one team.”*

-Researcher, Nepal

These challenges may have been mitigated with more time allocation dedicated to this type of E-data programming. One of the strengths of using a custom-built app was the flexibility to adapt and improve on the system within countries, and for users in line with design-thinking theory [51]. However, it was difficult to finalise the E-data app within this context. The pilot testing and feedback loops were an essential part of the development process but were also perceived to delay progress:

*“We did have feedback for the additional options in the variables, and had to ask the [app development] team to add the variables… It would take a long time to be updated.”*

-Researcher, Nepal

The transition from paper to E-data tool was complex especially because data collection tool design and variables could not be finalised ahead of coding the E-data tools:

*“To understand the paper-based [tool] and to implement [code it] in the application was difficult… Things could get lost in that transfer process if you were not careful.”*

-Researcher, Bangladesh

These experiences highlight an important conflict in the design process: flexibility is needed to evolve and advance tool design, however incorporating additional changes to the variable list and automatic skip patterns after they have been programmed is time consuming to implement.

Automated skip patterns were intended to enhance data quality and user-friendliness of the observation tool. However, more time for pilot-testing would have been useful as nuances in the configuration of some questions or skip patterns was lost. For example, recording “yes” or “no” that the fetal heart rate was auscultated, rather than the actual number of beats per minute that were heard. For frictionless feedback, we would recommend that either preliminary data collection is initiated in the same country as the app development team, with immediate data quality checking and ‘test’ analyses; or alternatively, experienced programmers are required as part of all site teams.

### Step 3: Recruitment and training of data collectors

Data collectors and supervisors required clinical training and were recruited on the basis of a written application, interview, and pre-employment testing regarding routine maternal and newborn health care. Candidates were also screened for previous E-data collection experience and competence using a smartphone. Data collectors received two weeks of training and needed to achieve ≥80% on post-training tests (Table 1).

**Table 1:** Data collector recruitment and training, EN-BIRTH study

	Bangladesh	Nepal	Tanzania
**Who were the trainers?**	EN-BIRTH research team: 7 Trainers from local hospital: 0 Other: 0	EN-BIRTH research team: 8 Trainers from local hospital: 3 Other: Head of department and hospital director were present during orientation.	EN-BIRTH research team: 14 Trainers from local hospital: 9 Other: 5 [administrators]
**Number of training participants**	Managers: 0 Supervisors: 4 Data collectors: 51 Total: 55	Managers: 4 Supervisors: 4 Data collectors: 27 Total: 31	Managers: 9 Supervisors: 12 Data collectors: 71 Total: 92
**Number of days for training**	Total: 11 days Theoretical: 7 days Hands-on: 4 days	Total: 2 weeks Theoretical: 7 days Hands-on: 7 days	Total: 2 weeks Theoretical: 7 days Hands-on: 7 days
**Pre-training test scores %**	Range: 25–85 Average: 60	Range: 16–87 Average: 52	Range: 15–82 Average: 45
**Post-training test scores %**	Range: 65–100 Average: 86	Range: 20–100 Average: 60	Range: 15–100 Average: 57
**Number who failed post-training tests**	2 Failed Extra training given and both eventually passed	4 Failed additional training was provided re-test was done and all were passed	14 Failed and did not proceed. Some observers were reallocated as trackers
**Additional training provision**	1 round, in 2 batches. Daily supervision and on the job training provided.	Daily supervision and on the job training provided.	On the job training where required. This was through monitoring and supportive supervision

The training programme covered EN-BIRTH study protocols, standard operating procedures, and induction on the E-data app. The component for observation on labour ward was adapted from the MCHIP Clinical Observer Learning Package curriculum used for a study in Mozambique [40] with reference to relevant DHS-7 survey modules. Training implementation was led by the country research teams with support from LSHTM. The training included the paper-based data collection tools (with emphasis on content), followed by tablet-care-and-use, hands-on data collection role plays using the EN-BIRTH app, classroom-based simulation training for responding to adverse or life-threatening events where hospital staff were not implementing local guidelines (Additional file 7), and field practise completing all four E-data capture tools [52]. The programme included one week of classroom based study and one week of hands-on practise in relevant clinical settings. One-to-one sessions and additional support were provided where necessary, and in Nepal, candidates had one opportunity to re-take the post-training testing if required (Table 1).

#### Respondents’ perspectives on recruitment and training of data collectors

Respondents reported that the training was sufficient, “most passed” (Table 1), and they appreciated the time to practice using the E-data app within clinical settings:

*“…some on the job training where it was necessary… helped keep everyone calm.”*

-Researcher, Tanzania

Materials and data collector tools were shared in the local language and all teams had flexibility to implement refresher training where needed:

*“We were in the wards with the data collectors… just helping them throughout the process.”*

-Researcher, Tanzania

The EN-BIRTH study collected a large number of variables, > 500 across four different tools within the E-data app. This was perceived as complex for data collectors, and respondents suggested more training focused on the five selected *Every Newborn* variables would have been helpful:

*“It would’ve been better if important indicators were prioritised while providing training. So many indicators sometimes [caused] confusion.”*

-Researcher Nepal

The E-data app included a feature for data collectors to record if health workers were observed to omit an intervention of interest, or if these data were missing however, the interpretation of these functions differed between hospitals. These challenges could have been addressed during training.

### Step 4: Data collection, quality assurance, and improvement

The EN-BIRTH E-data app contained built-in skip patterns, error messages, and rules to restrict data to realistic ranges and to monitor for data uniqueness or consistency, in addition to a data monitoring dashboard (Additional file 7). Data quality assurance procedures aimed to maintain the validity, accuracy, completeness, timeliness and reliability of data. Quality measures included implementation of the study protocol via standardised materials and training for all five EN-BIRTH hospitals, integrated E-data app quality-control features, hospital-based supervision of data collectors, tiered database and user-access appropriate to role and competence, pilot testing of paper-based and E-data research tools, and a unified variable matrix.

Data collection performance was reviewed via the web-based dashboard which provided a real-time summary of the *Every Newborn* coverage indicators of interest stratified by hospital, and a data capture cascade detailing the number of participants registered, consented, and the stage of data collection (started/completed: observation/ extraction/verification/survey). The dashboard included a traffic light system to indicate the overall progress for data collection by indicator using predefined thresholds and functionality to track performance by data collector, site, variable, and date (Fig. 3). The data dashboards were reviewed during fortnightly virtual meetings with representation from all four EN-BIRTH countries in addition to regular in country monitoring systems. This peer-to-peer collaboration and learning was central to identifying and solving challenges as they presented.

#### Respondents’ perspectives on data collection, quality assurance, and management

The E-data platform was perceived to improve the data collection processes in addition to data quality; especially with the implementation of the dashboard and bi-monthly multi-site meetings for data tracking and management:

*“Without the dashboard, [you] would have to go into the database every time to analyse and check if things were right. The beauty of collecting real time data, was that we had the database and could do some of the data monitoring virtually. We could also identify what possible mistakes teams or sites were making.”*

-Researcher, Bangladesh

Respondents provided numerous examples of collective problem solving including server management challenges, high staff turn-over, and pressure on data collectors to support with clinical work:

*“Nurses started asking, ‘why don’t you help me, you’re not doing anything? Why don’t you help me to document?”*

-Researcher, Nepal

This challenge was addressed via meetings with clinical managers, hospital staff and data collectors in all sites. Tanzania also pioneered roll-out of EN-BIRTH data collector uniforms (unique from that of the hospital staff); this idea was subsequently implemented in other EN-BIRTH hospitals. The team had systems in place for maintaining battery charge, availability of spare tablets, and repairing hardware locally where needed.

Some respondents felt that for interventions where the camera placement could capture the whole event without compromising ethical considerations, film evidence would have been useful for assessing inter-observer reliability:

*“On observation side, it’s really tricky making assurance on data quality. Filming would’ve been helpful, would’ve solved some issues where everything is happening at once.”*

-Researcher, Tanzania

Observations were terminated when participants were transferred out of the labour ward; this was problematic for assessing timing of interventions required within the first hour after birth, such as early initiation of breastfeeding, as many women were observed less than 1 h [53].

### Step 5: Data management, cleaning and analysis

Data entry was possible with or without internet connectivity and data were synchronised when connectivity was permitting. In the absence of internet access, data were stored on the tablet and uploaded once connectivity was reinstated. Once uploaded, data were stored on the country’s dedicated virtual or physical server. A local back-up schedule was implemented using either a separate server or external hard drive. Raw data were stored in an encrypted format, accessed only by country data managers and the E-data team. Data management procedures were standardised and included agreed protocols for database closure, export and server conservation, server decommissioning, anonymization of datasets, data transfer, renaming, merging and pooling, data quality assessments and data cleaning. The common database structure aimed to minimise data management errors, and excessive data backlogs. The variable matrix formed the basis for the EN-BIRTH code book, was disseminated to all members of the EN-BIRTH study team for topic specific analysis and write up. Data and para-data were available in several formats (Stata®, SPSS®, R®).

#### *Respondents’ perspectives on* management and analysis of data

Respondents found the flexibility of working on or offline essential, and appreciated opportunities for bi-lateral support between country teams to overcome challenges such as failure of the Nepal server.

*“Our server crashed down and that would have been a big problem. The support that came up was really good as we wouldn’t have been able to do [anything] otherwise.”*

-Researcher, Nepal

Overall, E-data capture was perceived to reduce data cleaning challenges, although there were several key learning opportunities:

*“We checked data once or twice a day and could talk with the supervisor if something was not working.”*

-Researcher, Bangladesh

Based on respondent’s experiences, we recommend that all time-stamped data entries should automatically include a date, and that open text options should be extensively pilot tested to improve efficiency and reduce data cleaning during analysis.

*“I found managing open text challenging. For example, there were hundreds of types of ceftriaxone… With many different spellings or brand names.”*

-Researcher, Bangladesh

## Discussion

This paper explores experiences of designing and implementing the E-data tool, which was custom-built for the EN-BIRTH study. EN-BIRTH was a large, observational study, assessing > 23,000 women and newborns in three countries, with unreliable internet connectivity. While E-data platforms are increasingly available and implemented within study settings and as part of routine data collection, there are few papers describing the experience of data collection and implementation, especially using customised or novel E-data platforms for complex clinical observation. Whilst our paper applied the process to a research study, the choices and learning are also relevant to design and use of E-data systems in many LMICs [54, 55].

Simultaneous capture of multiple, complex maternal and newborn health interventions, was considered essential by all team members in designing the EN-BIRTH E-data app. Direct data capture addresses several data quality challenges found with paper-based tools, avoiding data collectors having to flip through pages to follow skip patterns [19, 21–23, 29]. These issues have been described primarily for survey tools [56]. E-data collection has been implemented for intrapartum observation in several studies, although the experiences of use were not reported [10, 11, 17]. We found the opportunity to customise both the E-data interface, and automate skip patterns was imperative for observation of potentially concurrent events during labour and birth by one observer per participating woman. This was in contrast to a study in Tanzania that reports E-data collection tools enabled data collectors to observe up to three births simultaneously [10].

Whilst the EN-BIRTH E-data platform offered flexibility to ensure design was appropriate to the task and context, it is difficult to implement structural change in custom-built E-data tools once they have been programmed [29]. Extensive pilot testing of paper tools, as well as early versions of the E-data tool, are therefore imperative but increase the time investment and so have associated financial implications. We recommend planning for time (including contingency), to accommodate an iterative testing process, to avoid challenges of major revisions in E-data tools once they are programmed. This is especially important for programme contexts making the shift from paper to E-data capture [57, 58].

There are a range of E-data tools available within the public domain [37, 38, 59–62] (Additional file 6). For studies with less complexity, use of an existing customisable E-data capture platform may prove more cost effective, while still benefiting from E-data advantages such as direct and faster data capture, and real-time quality controls [19, 28, 63]. For example, a cohort study in Pelotas, Brazil found that using REDCap enabled researchers to collect 1243 additional variables with no increase in data collection time [19]. There is growing evidence to suggest that despite higher initial implementation costs, these efficiencies can lead to significant savings, especially for larger studies [19, 20, 23]. For large clinical trials, modelling suggests that cost savings gained from efficiencies in work load with reduced error and query rates, could equate to savings of 49 to 62% compared with paper-based data collection [20].

Despite standardised training in all sites for the E-data tool, we found implementation differences between countries. For example, how teams applied the options of “not observed” and “not done” when observing in the labour ward. These findings may also be relevant for studies using customisable smartphones software [9, 10, 17], such as Mobile data studio [64]. Multiple open text fields and data captured in four different languages requiring translation, were time consuming to clean (as required translation and back checking), therefore thorough pilot testing for open text options is also recommended, and especially pertinent to programme settings where human resources are often limited [57]. We also recommend that the piloting phase include implementation of “test” analysis on samples for key indicators, with calculation of Cohen’s Kappa coefficients for a set of duplicate observations.

Variable internet connectivity was a major consideration in the design of EN-BIRTH E-data software, and may be even more challenging for rural survey data collection [30]. Poor internet connection is a significant challenge in many LMIC settings [65], and our experiences highlight the necessity of tablet and server back-up systems in such contexts [28, 66]. Our tool supported data collection on and offline, and afforded flexibility in the choice of server. This had implications for live linking of case records throughout the different stages of the study, and for data quality monitoring which all required connectivity. High-volume data transmission requirements and inconsistent connectivity meant that some data were lost before reaching the server. This was particularly problematic if data collectors wanted to reassign their open case at the end of their shift, which required synchronisation between tablets and the server. Given intrapartum care transcends routine working periods with women admitted during labour and birth for many hours, the E-data tool was designed to accommodate shift changes between data collectors. Although this function was extremely useful, disruptions to the internet connection culminated in permanent data loss for some cases. The EN-BIRTH study team also overcame complete server malfunction in Nepal. Adherence to the data management procedures meant that disruption to data collection and loss of data were minimal (Additional file 9). While there are several other studies using E-data tools for observation of intrapartum events [9–11, 17], there is little published information exploring how these challenges were addressed.

Accessibility of data for dashboards and intermediary quality checking was a key advantage allowing early identification and course correction of issues [19, 20, 29, 56]. Other studies have reported complex dashboards are often underused [26, 30]. Indeed, a key challenge for the implementation of digitalised HMIS are the pluralistic approaches to design and content, which contribute to fragmented systems, over complexity in tools and potentially less comparable data [67].

Direct data capture provides increased security, and avoids some logistics transporting checklists, surveys, and managing photocopies and printing [21, 56], these advantages could be particularly pertinent in programme settings [65]. The EN-BIRTH team were comfortable using the tablets and had successful systems in place for maintaining battery charge, availability of spare tablets, and repairing hardware locally where needed. This was a hospital-based study, and different constraints may be presented for field work in remote or rural areas with no power supply [29, 30, 56]. Choice of hardware was evaluated within the individual local contexts during the formative research phase and the EN-BIRTH E-data team supported with maintenance of hard and software throughout; success relies on high levels of trust and communication between participating institutions and partners. Opportunities for peer to peer collaboration and learning were highly valued by the EN-BIRTH team and we recommend instituting these mechanisms in the early phases of study design. Within programme settings, this highlights the importance of adopting a user-focused design approach and ensuring the inclusion of all major stakeholders [54, 67].

### Strengths and limitations

EN-BIRTH included five hospitals from three LMICs, so our experiences and learning are likely to be relevant for studies facing similar connectivity challenges and resource limitations. Descriptive data are based on meeting notes, study protocols, operating procedures, and email correspondence as this paper is outside the primary study objectives. The absence of a reference method impeded any opportunity to compare the EN-BIRTH E-data tool with paper-based or digital alternatives. Qualitative data were drawn from a selection of research team members in all participating countries, however, four invitees were unable to join, and data collectors were not interviewed who may have bought a different perspective. Given all participants contributed to the design and inception of the E-data tool, there is a risk of reporting bias favourable to the tool. It was difficult to assess if saturation was met given the small sample size, however we have circulated this manuscript to the EN-BIRTH study group for their inputs and comments. We have also compared our findings with evidence from the current literature to identify and discuss unusual results. Assessment of the cost effectiveness would have been useful and we hope the E-data tool can be easily adapted in service of other observation studies.

## Conclusions

The custom-built E-data tool was perceived as valuable for collecting observation data for the core purpose of EN-BIRTH, with observation of rapid, concurrent maternal and newborn events during labour and birth. The app interface, time-stamping function, and automated skip patterns were user-friendly. Poor internet connection is a significant challenge in many LMICs and could compromise transmission of high-volume data without proper management. We found direct data capture had potential for improving data quality, but only with careful planning, which can be time consuming. We would recommend extensive pilot testing of tools to ensure accurate transition between paper and electronic formats, and to double check skip patterns. Ongoing data supervision is key for collector proficiency post training. Consideration of the purpose (for study or programme), the alternatives, and the costs are important before committing to a custom-built tool.

## Supplementary Information

**Additional file 1.** EN-BIRTH timeline and data collection dates by site, EN-BIRTH study.

**Additional file 2.** Focus group discussion guide on EN BIRTH data collection.

**Additional file 3.** Consolidated criteria for reporting qualitative research (COREQ) checklist.

**Additional file 4.** Ethical approval by local institutional review boards, EN-BIRTH study.

**Additional file 5.** Data flow assessment checklist by EN-BIRTH intervention.

**Additional file 6.** Overview of existing electronic data collection tools and platforms.

**Additional file 7.** Key features of the EN-BIRTH data capture application.

**Additional file 8.** Android tablet readiness assessment, EN-BIRTH study.

**Additional file 9.** Data management and server maintenance user checklist, EN-BIRTH study.

## Data Availability

The datasets generated during and/or analysed during the current study are available on LSHTM Data Compass repository, https://datacompass.lshtm.ac.uk/955/.

## Abbreviations

COREQ: Consolidated Criteria for Reporting Qualitative Research
CEmONC: Comprehensive emergency obstetric and newborn care
CIFF: Children’s Investment Fund Foundation
DHS: The Demographic and Health Surveys Program
E-data: Electronic data
E-data app: EN-BIRTH custom-built android tablet-based electronic data capture system
EN-BIRTH: *Every Newborn*-Birth Indicators Research Tracking in Hospitals study
FGD: Focus Group Discussion
icddr,b: International Centre for Diarrhoeal Disease Research, Bangladesh
KMC: Kangaroo mother care
LMIC: Low and Middle Income Country/Countries
LSHTM: London School of Hygiene and Tropical Medicine
MCHIP: Maternal and Child Health Integrated Programme
MICS: Multiple Indicator Cluster Surveys
WHO: World Health Organization
