# Supplementary material for: Electronic data collection for multi-country, hospital-based, clinical observation of maternal and newborn care: EN-BIRTH study experiences
Source: BMC Pregnancy Childbirth. 2021 Mar 26;21(Suppl 1):234. doi: 10.1186/s12884-020-03426-5 (PMC7995708; doi:10.1186/s12884-020-03426-5)
Supplement: Supplementary file 1 — Additional file 1. EN-BIRTH timeline and data collection dates by site, EN-BIRTH study. [file 12884_2020_3426_MOESM1_ESM.pdf]

SUPPLEMENT TITLE: Every Newborn BIRTH multi-country validation study: informing measurement of coverage and quality of maternal and newborn care
PAPER TITLE: Electronic data collection for multi-country, hospital-based, clinical observation of maternal and newborn care: EN-BIRTH study experiences

Additional file 1: EN-BIRTH timeline and data collection dates by site, EN-BIRTH study

Figure 1. EN-BIRTH timeline

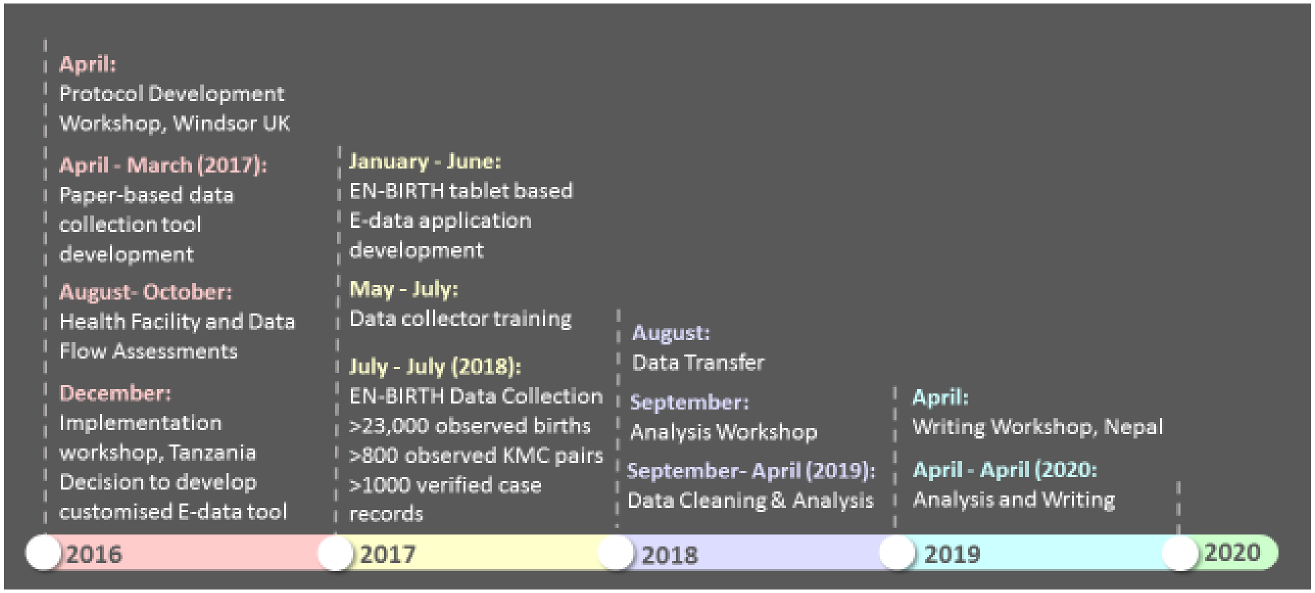

Table 1. Data collection dates by site

EN-BIRTH study	Bangladesh		Nepal	Tanzania		All sites
	Azimpur Tertiary	Kushtia District	Pokhara Regional	Temeke Regional	Muhimbili National	
**Tablet data collection dates**	17/8/17 to 30/4/18	11/7/17 to 30/5/18	17/7/17 to 31/7/18	3/7/17 to 30/5/18	3/7/17 to 28/2/18	
Duration	8 months	10 months	1 year	10 months	7 months	
Original register used:	17/8/17 to 18/10/17	25/8/17 to 27/9/17 (due to short supply)	Not applicable	Not applicable	Not applicable	
Revised register used:	19/10/17 to 30/4/18	11/7/17 to 24/8/17 and 28/9/17 to 30/5/18	Not applicable	Not applicable	Not applicable	
**Register extraction comparison dates to assess biases**						
**Pre-study**	1/1/16 to 31/12/16	1/1/16 to 31/12/16	1/4/16 to 31/3/17	1/1/16 to 31/12/16	1/1/16 to 31/12/16	
Duration	12 months	12 months	12 months	12 months	12 months	
**During/after-study**	17/8/17 to 17/8/18	11/7/17 to 11/7/18	17/7/17 to 17/7/18	3/7/17 to 3/7/18	3/7/17 to 3/7/18	
Duration	12 months	12 months	12 months	12 months	12 months	
**Time elapsed between delivery and exit survey interview/ days**	n (%)	n (%)	n (%)	n (%)	n (%)	n (%)
**Total**	2844	2331	6922	5752	2783	20632
0-1 day	725 (25.5)	1345 (57.7)	5854 (84.6)	5433 (94.5)	1009 (36.3)	14366 (69.6)
2-3 days	511 (18)	846 (36.3)	833 (12)	181 (3.1)	1098 (39.5)	3469 (16.8)
4+ days	1599 (56.2)	127 (5.4)	154 (2.2)	43 (0.7)	597 (21.5)	2520 (12.2)
Missing	9 (0.3)	13 (0.6)	81 (1.2)	95 (1.7)	79 (2.8)	277 (1.3)
Mean	3.1	1.3	0.6	0.7	3.2	1.4
Median	4.0	0.0	0.0	0.0	2.0	1.0
