# Supplementary material for: Electronic data collection for multi-country, hospital-based, clinical observation of maternal and newborn care: EN-BIRTH study experiences
Source: BMC Pregnancy Childbirth. 2021 Mar 26;21(Suppl 1):234. doi: 10.1186/s12884-020-03426-5 (PMC7995708; doi:10.1186/s12884-020-03426-5)
Supplement: Supplementary file 2 — Additional file 2. Focus group discussion guide on EN BIRTH data collection. [file 12884_2020_3426_MOESM2_ESM.pdf]

SUPPLEMENT TITLE:
Every Newborn BIRTH multi-country validation study: informing measurement of coverage and quality of maternal and newborn care

PAPER TITLE:
Electronic data collection for multi-country, hospital-based, clinical observation of maternal and newborn care: EN-BIRTH study experiences

Additional file 2: Focus group discussion guide on EN BIRTH data collection

To discuss experiences from research team using the EN-BIRTH customized tablet/ app based data capture system

Focus Group Discussion / Key Informant Interviews

Participants:

1. **Data Managers (in country (BD) and remote (NP and TZ)**
2. Researchers (PIs and other team members)
   Have provided written feedback on findings instead
3. Data supervisors
   Not included as moved on at time of interviews

Aim:
To share experiences from field/sites regarding the EN-BIRTH tablet/ app-based data capture system and suggest ways to improve for future use
The EN-BIRTH data collection tool has 3 distinct parts and good to include all 3 throughout the discussions.

1. Observation green/red/grey/white button section
2. Register Data Extraction
3. Exit survey interviews

Please explain to participants that the objective of this FGD is to share experiences (barriers, enablers and recommendations) regarding the use of EN-BIRTH tablet/ app based data capture system.

''If you agree to take part, you will join a focus group discussion via Zoom conferencing. Participation in this study will not inflict any harm on you.
All information collected about you during will be kept strictly confidential and not shared with anyone outside the study team. The data will be coded so that the personal identity and individual data from observations are traceable only with the code key which will be held by the study researchers, no one else will have access to it. We hope to publish results as part of an overall paper reflecting the choices and lessons learned from the EN-BIRTH journey in a scientific journal. The study is completely voluntary and you are entitled to withdraw at any point without giving any explanation about your decision. If you decide to leave the study, any data (whether it is a partial or complete collection of data) that refers specifically to you will be destroyed and researchers will not be allowed to use it in this study or future studies. This will not impact on any professional relationships or opportunities.''

The FGD will look at these themes/areas for discussion organized by 5 process steps and one general question:

Step 1 Selection of EN-BIRTH study data collection approach and software
Step 2 Design and programming data collection tools
Step 3 Recruitment and training of Data collectors
Step 4 Data collection, quality assurance, and management
Step 5 Management and analysis of data
General question: Recommendations for its improvement for future use

No right or wrong answer. We are here to share experiences.
Background information from each site:

Name of study country	BD/ NP/ TZ	Remarks/ Notes
# of staff involved in FGD		
Date and time of interview for FGD		
Name of moderator		
Name of notes taker		

Step 1: Selection of software approach for EN-BIRTH data collection
Here we will discuss and share experiences on how the tablet based data collection approach was established across sites.

1.1 What are the differences between paper based and app based data collection?
1.2 Why do you think EN BIRTH study selected app based data collection for this study? (probe- Tanzania workshop)

Step 2 Design and programming data collection tools
Here we will ask about your experiences about finalizing the tools and contents to integrate into the app based platform and set up the server

2.1 What were the content development stages and how did you go through the process? (Probe- data flow, desk review of the register data, finalization of the variables)
2.2 Can you share your experiences of using the variable matrix? Did you find it helpful or challenging to use?
2.3 Where did you field test the app? How long it took? What kind of feedback and recommendation you provided for the app?
2.4 How were your recommendations received?
How many changes/ versions were made before coming up with a stable version?
(Probe- trouble shooting, regular update)
2.5 Tell us experiences to set up the server in your country? Did you face challenges or difficulties?

Step 3 Recruitment and training of Data collectors

Here we will discuss and share experiences on how the training for the data collectors went across sites.
Collect information for below table first-
Who were the trainers?
# of data collectors trained
# of data collectors who had previous knowledge on ever using a tablet
# of Supervisors/ managers trained
# of days for training
% of participants passed the assessment (base on pretesting and post-testing)
# of refresher training conducted
# of tablet used for the data collection

Then answer the following questions-

3.1 Were the days for training adequate?
3.2 Can you share some of your experiences from the training: probe: What did you like and/or dislike? How the training could have been improved?
3.3 Were there any challenges with the training or demonstration of app? How were the challenges overcome?

Step 4 Data collection, quality assurance, and management

4.1 Can you tell me about the experience/ challenges of using tablet app to collect data in the study sites
(Probe- ask participants to share both positive and negative experiences- for tracking, observations, recall surveys, verification and data extraction)?
How were the challenges overcome?
4.2 Did you use any paper based notes or other aides to support your data collection with the app or when the app stopped working? If yes, how was it done?
4.3 How was monitoring of the data collection performed? Describe the process
(Probe- dashboard, supervisor monitoring using tablet, skype call with all sites) –
any suggestions for improvement?
4.4 How did you came up with the finalization of variables that were displayed in dashboard?
4.5 Did you find the dashboard useful or not
(Probe- completion rate, colour changing)?
How did you check data quality?
(Probe- real time data monitoring and its implication)
4.6 Did you use other methods than the app to monitor progress and quality? Any recommendations for future use?

Step 5 Management and analysis of data

We will ask about their experiences from the field and how they overcame those challenges.

5.1. Can you please tell us about the quality of dataset that was obtained using the application?
5.2. How much time you invested for data cleaning and data management?
5.3. Tell us about the challenges that you faced in terms of data uploading and data security issues. (Probe- data loss, server crash)
5.4. Any suggestions to improve the data uploading system and security concern (Probe- load shedding issues, internet problem, sync problem)
5.5. Data management support

General question:
General recommendations for app based data collection

6.1. Would you recommend to use in future research studies?
    -EN-BIRTH study tool itself
    -A similar tool based on a similar design
    (ask for- Observation, Register extraction, exit interviews)
6.2. Why/Why not?
6.3. If we are to do data collection again, what will be your recommendations to the field team?

Thank you for your time and the information shared. Do you have any questions?
Moderator/Notes taker's comments and observation:
