# Supplementary material for: Electronic data collection for multi-country, hospital-based, clinical observation of maternal and newborn care: EN-BIRTH study experiences
Source: BMC Pregnancy Childbirth. 2021 Mar 26;21(Suppl 1):234. doi: 10.1186/s12884-020-03426-5 (PMC7995708; doi:10.1186/s12884-020-03426-5)
Supplement: Supplementary file 3 — Additional file 3. Consolidated criteria for reporting qualitative research (COREQ) checklist. [file 12884_2020_3426_MOESM3_ESM.pdf]

SUPPLEMENT TITLE:
Every Newborn BIRTH multi-country validation study: informing measurement of coverage and quality of maternal and newborn care

PAPER TITLE:
Electronic data collection for multi-country, hospital-based, clinical observation of maternal and newborn care: EN-BIRTH study experiences

Additional file 3: Consolidated criteria for reporting qualitative research (COREQ) checklist

**Topic**		**Guide Questions/ Description**	**Report**
**Domain 1: Research team and reflexivity**			
Personal Characteristics			
Interviewer/facilitator	1	Which author conducted the focus group?	HR, SK
Credentials	2	What were the researchers qualifications	HR: BSc, MSc, PGCILT, RM.<br>SK: BSc, MSc
Occupation	3	What was their occupation at the time of the study	Research Fellow, research assistant.
Gender	4	How did they identify?	Females
Experience and Training	5	What experiences or training did they have?	HR: 5 years research experience with the EN-BIRTH study. SK: no prior research experience.
Relationship with Participants			
Relationship established	6	Was the relationship established before the study?	HR: Yes<br>SK: No
Participant knowledge of interviewer	7	What did the participants know about the researcher?	HR: Participants and Researcher are colleagues and share insight into the researchers bias, assumptions, reasons and interests.<br>SK: no prior relationship.
Interviewer characteristics	8	What characteristics were reported about the facilitator?	The researcher was part of the EN-BIRTH research team and also contributed to design of the E-data app. All participants were on a comparable or higher level of the organigram in terms of

			managerial structure and none of the participants were line managed by the researcher.
**Domain 2: Study Design**			
Theoretical Framework			
Methodological orientation	9	e.g. Grounded theory, discourse analysis, ethnography, phenomenology, content analysis	Content analysis
Participant Selection			
Sampling	10	How were participants selected?	Purposive sample
Method of approach	11	How were participants approached? e.g. face-to-face, telephone, mail, email	They were approached via email
Sample Size	12	How many participants were in the FGDs	10 (8 participants and 2 researchers)
Non-Participation	13	How many people did not accept invitation to participate	4
Setting			
Setting of data collection	14	Where were the data collected?	At participant's work place
Presence of non-participants	15	Was anyone else present outside the participants and the researchers?	No
Description of sample	16	What are the important characteristics of the sample?	The sample included three EN-BIRTH data managers, one co-principle investigator, and four study implementers who were also involved in data analysis. Two of the participants also had experience of coding the new software.
Data Collection			
Interview Guide	17	Were questions, prompts, guides provided by authors? Were they pilot tested?	Additional File 2
Repeat interviews	18	Were repeat interviews carried out?	No
Audio visual recording	19	Did the researchers use audio or visual recording to collect data?	Yes- Audio recording and transcription in English.

Field notes	20	Were field notes made?	No
Duration	21	What was the duration of the interviews or focus group?	105-120 minutes
Data saturation	22	Was data saturation discussed?	Yes- Unable to assess if data saturation occurred because limited opportunity to undertake interviews, however, the paper was distributed to all team members for their comments/ corrections.
Transcripts returned	23	Were transcripts returned to participants to comment on or correct?	Yes
**Domain 3: analysis and findings**			
Data analysis			
Number of data coders	24	How many data coders coded the data?	1- HR (checked by SK).
Description of the coding tree	25	Did authors provide a description of the coding tree?	Yes.
Derivation of themes	26	Were themes identified in advance or derived from the data?	Data was coded according to the five pre-defined steps, and emergent themes coded within these sub-categories.
Software	27	What software, if applicable, was used to manage the data?	NVIVO
Participant checking	28	Did participants provide feedback on the findings?	Yes
Reporting			
Quotations reported	29	Were participant quotations presented to illustrate the themes/findings? Was each quotation identified?	Yes for both
Data and findings consistent	30	Was there consistency between the data presented and the findings?	Yes.
Clarity of major themes	31	Were major themes clearly presented in the findings?	Yes- structured according to the 5 steps.

Clarity of minor themes	32	Is there a description of diverse cases or discussion of minor themes?	Yes- within each step, sub themes are also presented and discussed.
