# Supplementary material for: Electronic data collection for multi-country, hospital-based, clinical observation of maternal and newborn care: EN-BIRTH study experiences
Source: BMC Pregnancy Childbirth. 2021 Mar 26;21(Suppl 1):234. doi: 10.1186/s12884-020-03426-5 (PMC7995708; doi:10.1186/s12884-020-03426-5)
Supplement: Supplementary file 5 — Additional file 5. Data flow assessment checklist by EN-BIRTH intervention. [file 12884_2020_3426_MOESM5_ESM.pdf]

SUPPLEMENT TITLE:
Every Newborn BIRTH multi-country validation study: informing measurement of coverage and quality of maternal and newborn care

PAPER TITLE:
Electronic data collection for multi-country, hospital-based, clinical observation of maternal and newborn care: EN-BIRTH study experiences

Additional file 5: Data flow assessment checklist by EN-BIRTH intervention

COUNTRY, SITE:

Antenatal Corticosteroids:
1. List of registers (and special registers) and items used to monitor indicators related to this intervention
2. Please attach the registers (if available)
3. Who usually fills in the register(s), if known

Neonatal Resuscitation:
1. List of registers (and special registers) and items used to monitor indicators related to this intervention
2. Please attach the registers (if available)
3. Who usually fills in the register(s), if known

Treatment of inpatient neonatal infections:
1. List of registers (and special registers) and items used to monitor indicators related to this intervention
2. Please attach the registers (if available)
3. Who usually fills in the register(s), if known

Kangaroo Mother Care:
1. List of registers (and special registers) and items used to monitor indicators related to this intervention
2. Please attach the registers (if available)
3. Who usually fills in the register(s), if known

Uterotonics for prevention of postpartum haemorrhage:
1. List of registers (and special registers) and items used to monitor indicators related to this intervention
2. Please attach the registers (if available)
3. Who usually fills in the register(s), if known

Please provide the schematic representation of the data flow in each facility, reflecting:
- Creation of admission record
- Points where data is recorded for each intervention
- Links between registers (if any)
- Links between registers and patient record
- Who fills in the registers and when
- Is all data aggregated into one at the ward/department/hospital level?

Please provide notes, if possible, when the data is being entered (right after the intervention, end of the shift, same day, end of the week, other [specify]? Who is responsible for data aggregation? Is this data being used by hospital management/decision-making? Is data being checked and verified for completeness? Are there data quality check in place? Specify which one? Is data used for reports generation, if so, which indicators? Is data further submitted to regional/state level?
