# Supplementary material for: Electronic data collection for multi-country, hospital-based, clinical observation of maternal and newborn care: EN-BIRTH study experiences
Source: BMC Pregnancy Childbirth. 2021 Mar 26;21(Suppl 1):234. doi: 10.1186/s12884-020-03426-5 (PMC7995708; doi:10.1186/s12884-020-03426-5)
Supplement: Supplementary file 6 — Additional file 6. Overview of existing electronic data collection tools and platforms. [file 12884_2020_3426_MOESM6_ESM.pdf]

SUPPLEMENT TITLE:
Every Newborn BIRTH multi-country validation study: informing measurement of coverage and quality of maternal and newborn care

PAPER TITLE:
Electronic data collection for multi-country, hospital-based, clinical observation of maternal and newborn care: EN-BIRTH study experiences

Additional file 6: Overview of existing electronic data collection tools and platforms

TOOL / PLATFORM	KEY FEATURES RELEVENT TO THE EN-BIRTH STUDY
**Considered for EN-BIRTH**	
**KoBo Toolbox**	• Type of data collection: Survey<br>• No license purchasing required (free), open source<br>• Data collection online and offline<br>• Compatible for use with phones, tablets, & any browser<br>• Security: data synchronized via SSL to protect data<br>• Quality monitoring: data immediately available<br>• Visualization: graphs, tables, maps with disaggregation.<br>• Export all your data at any time<br>• Supported formats: Excel, CSV, KML, ZIP (for media) and SPSS<br>• Access your data through API<br>• Source: https://www.kobotoolbox.org/
**Open Data Kit**	• Type of data collection: survey<br>• No license purchasing required (free), open source<br>• Data collection is offline.<br>• Compatible for use with phones, tablets<br>• Security: offline<br>• User support available, including user forum but adaptation of the questionnaire requires strong coding skills<br>• Displays one question on a page<br>• Doesn't support creation/displaying complex tables<br>• Source: https://getodk.org/
**Other Available E-data Tools**	
**Census and Survey Processing System (CSPro)**	• Type of data collection: survey<br>• No license purchasing required (free), open source<br>• Data collection online and offline<br>• Compatible for use with phones, tablets, & any browser<br>• Security: robust security systems<br>• User support: limited<br>• Quality monitoring: data immediately available<br>• Visualization: graphs, tables, maps with disaggregation.<br>• Displays one question on a page<br>• Adaptation of the questionnaire for study requires strong coding skills and knowledge of ASCII language<br>• Source: https://www.census.gov/data/software/cspro.html

**Epi Info**<br><br>CDC, Atlanta, GA	• Type of data collection: Survey<br>• No license purchasing required (free), open source<br>• Data collection online and offline<br>• Compatible for use with phones, tablets, & any browser<br>• Security: robust security systems<br>• Quality monitoring: data immediately available<br>• Visualization: graphs, tables, maps with disaggregation.<br>• User support: easy setup, customizable forms<br>• Access: http://www.cdc.gov/epiinfo
**REDCap**<br><br>Vanderbilt University	• Type of data collection: any type of data, including in environments compliant with electronic records USA legislation<br>• No license purchasing required (free), open source<br>• Data collection online and offline<br>• Compatible for use with phones, tablets<br>• Security: robust security systems<br>• Quality monitoring: data immediately available<br>• Visualization: graphs, tables, maps with disaggregation.<br>• User support: limited, need strong coding skills and knowledge of this system<br>• Access: http://www.project-redcap.orgexternal icon
**Survey solutions**<br><br>World bank	• Type of data collection: survey<br>• No license purchasing required (free), open source<br>• Data collection online and offline<br>• Compatible for use with phones, tablets, & any browser<br>• Security: robust security systems<br>• User support: minimal technical and programming skills required for survey adaptation and survey coding.<br>• Quality monitoring: data immediately available<br>• Visualization: graphs, tables, maps with disaggregation.<br>• Access: http://surveys.worldbank.org/capi
