# Supplementary material for: Electronic data collection for multi-country, hospital-based, clinical observation of maternal and newborn care: EN-BIRTH study experiences
Source: BMC Pregnancy Childbirth. 2021 Mar 26;21(Suppl 1):234. doi: 10.1186/s12884-020-03426-5 (PMC7995708; doi:10.1186/s12884-020-03426-5)

SUPPLEMENT TITLE:
Every Newborn BIRTH multi-country validation study: informing measurement of coverage and quality of maternal and newborn care

PAPER TITLE:
Electronic data collection for multi-country, hospital-based, clinical observation of maternal and newborn care: EN-BIRTH study experiences

Additional file 7: Key features of the EN-BIRTH data capture application

Structure: Controls used in the EN-BIRTH E-Data collection Application
In the EN-BIRTH E-Data application, some input controls were used for the data collection. These are described below.

Command button and colour coding

- Command button allows the data collector in capturing a single response from multiple responses
- Color-coding allows the data collector to identify a response

Colour	Used in	Description
Green	OBS, MRS, DE, ACS, VER	When required action is completed,/Observed-Done
Red	OBS, MRS, DE, ACS, VER	When action is incomplete / Observed-Not Done
Orange	Participant	Participant switching button
White	Observation Module	Don’t know
Yellow	Observation Module	Selected Tab

Check Box and Radio button:

- Check boxes allow the data collector to capture multiple responses
- Radio button allows the data collector to select one among the multiple response

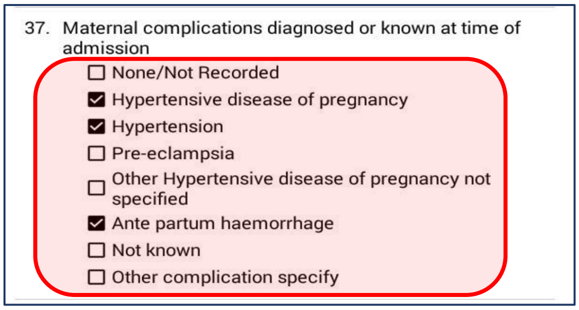

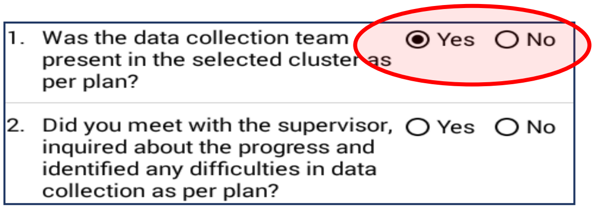

Drop Down List:

- Provides multiple choices from a list of mutually exclusive values. However, data collectors are able to select only the most appropriate option.
- If data collector touches the Drop Down control, a list will be available from where s/he can select the answer/response

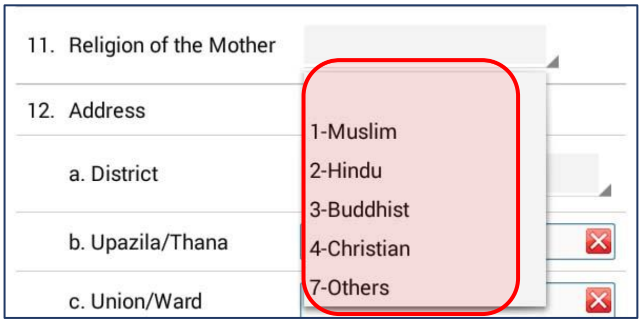

Text Box:

- First tap to Text Box will make the button active and the APP keyboard (for letter) or keypad (for number) will appear in front of you. Fill up the Text Box by writing appropriate answer.

Calendar and Clock:

- Records the appropriate date and time using the APP Calendar and APP Clock.

DIFFERENT USER AND TYPES OF DATA COLLECTION

Users:

Different types of user access were programmed into the EN-BIRTH E-data App. Each type of user was assigned relevant access for their given activities and data collection. Each user was given a User ID (with a password) to log in into the ENAP system.

- Tracker
- Observer
- Interviewer
- Data Extractor and Verifier
- Supervisor
- ''Super user'' / member of the E-data app development team

Registration and Tracking:

Tracker can register patient for Labour and delivery, kangaroo mother care and neonatal infection by clicking the button of the same name.

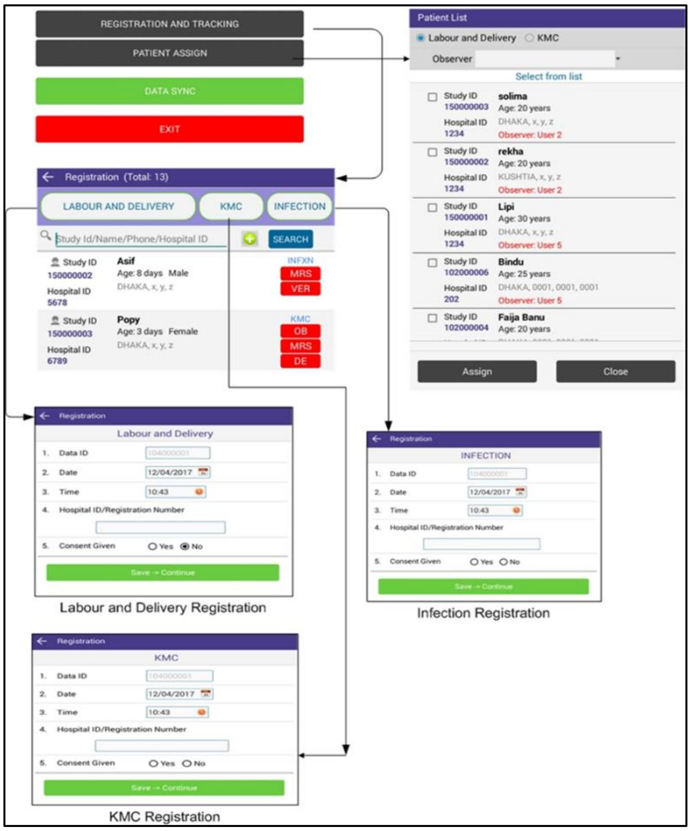

We have also incorporated another two buttons in the Labour and Delivery observation window- Pause button and Stop button

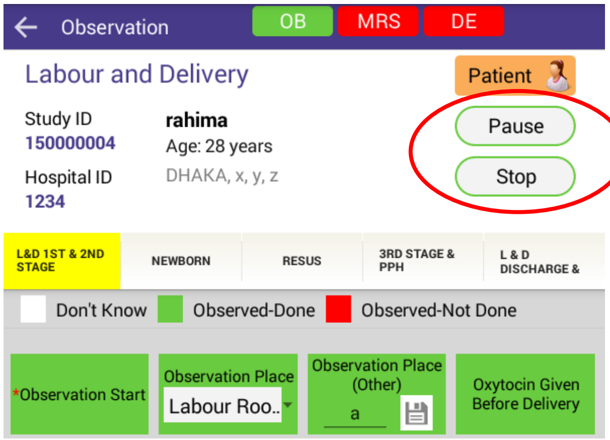

Pause button:

An observer was able to pause the observation at any stage of observation by clicking the PAUSE button. Clicking on pause, a pop-up window appears for confirmation.

Stop button:

Due to some unavoidable circumstances (clinical incidents, revoke of consent etc.), an observer could STOP the observation at any stage by clicking the STOP button. By using this command, an observer can pre-maturely end the observation.

Standard operating procedure for life threatening events where no appropriate action is being observed as initiated:

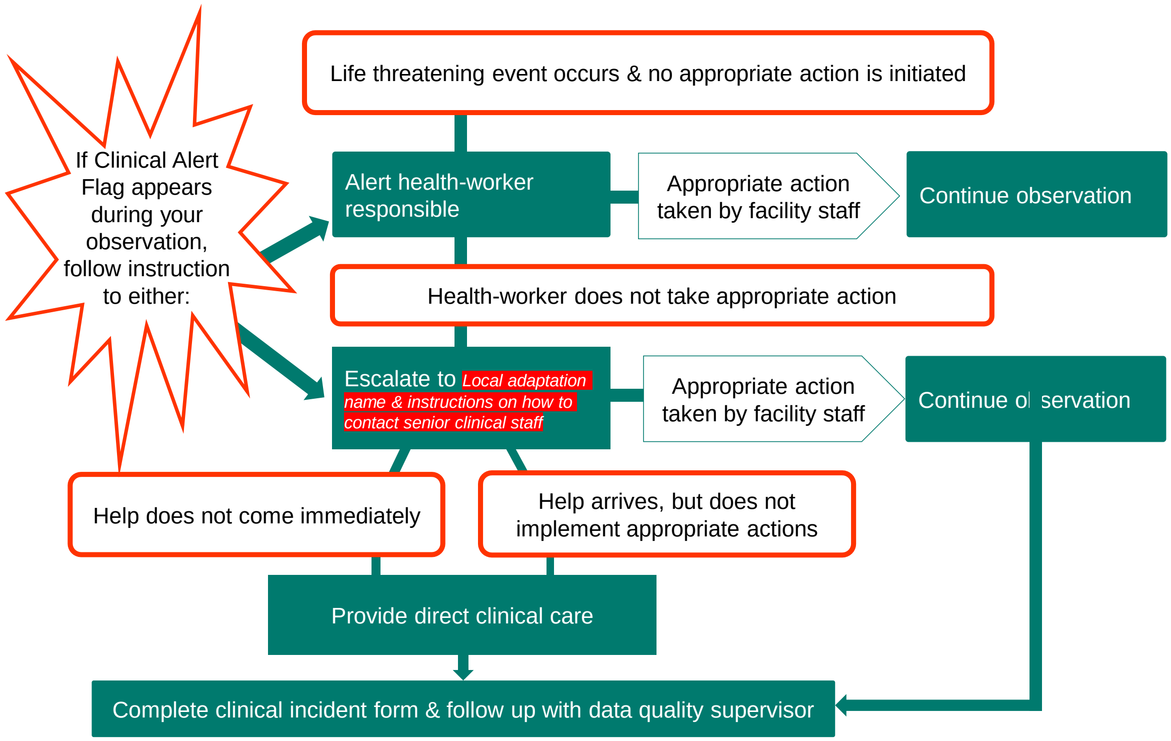

Supplement: Supplementary file 7 — Additional file 7. Key features of the EN-BIRTH data capture application. [file 12884_2020_3426_MOESM7_ESM.pdf]
