# Supplementary material for: Electronic data collection for multi-country, hospital-based, clinical observation of maternal and newborn care: EN-BIRTH study experiences
Source: BMC Pregnancy Childbirth. 2021 Mar 26;21(Suppl 1):234. doi: 10.1186/s12884-020-03426-5 (PMC7995708; doi:10.1186/s12884-020-03426-5)
Supplement: Supplementary file 8 — Additional file 8. Android tablet readiness assessment, EN-BIRTH study. [file 12884_2020_3426_MOESM8_ESM.pdf]

SUPPLEMENT TITLE:
Every Newborn BIRTH multi-country validation study: informing measurement of coverage and quality of maternal and newborn care

PAPER TITLE:
Electronic data collection for multi-country, hospital-based, clinical observation of maternal and newborn care: EN-BIRTH study experiences

Additional file 8: Android tablet readiness assessment, EN-BIRTH study

	Bangladesh	Nepal	Tanzania
**Total number of facility based research staff**	Original training = 55<br>Total remaining = 55	Original training = 31<br>Total remaining = 35	Original training = 92<br>Total remaining = 65<br>(33 Temeke, 32 Muhimbili)
**Available Tablets**	>300	0	2
**Tablets to be procured**	16 (for observation)	18	19
**Brand and screen size**	Samsung 7” Tab (available)<br>Lenovo 10” Tab (to be procured)	Samsung Tab A10”<br>and Samsung 7”	Samsung 7"
**Platform, version**	Android 4.1 or above	Android 4.1 or above	4.0 and above
**Previous experience with Android app development**	Developed application for Demographic Surveillance (one of them covers above 650,000 Population), Hospital surveillance, Survey, Call in Centre on Android platform.	None	Using ionic 2 framework
**Previous Software Experience**	Software: Android Studio, JAVA; Monitoring software: Web Application: ASP .NET code behind C#, HTML	KoBo Toolbox<br>Census and Survey Processing System(CSPro)	Open Data Kit
**Software used for data management**	Database in Mobile device: SQLite; Database Server: SQL Server 2008 R2; Data checking/Analysis: STATA 13/ SPSS 17	SPSS 17	Open Data Kit aggregate<br>National health management information system E-data tools
**Location of Server**	Local	Local	Local and<br>Cloud
