# Supplementary material for: Electronic data collection for multi-country, hospital-based, clinical observation of maternal and newborn care: EN-BIRTH study experiences
Source: BMC Pregnancy Childbirth. 2021 Mar 26;21(Suppl 1):234. doi: 10.1186/s12884-020-03426-5 (PMC7995708; doi:10.1186/s12884-020-03426-5)
Supplement: Supplementary file 9 — Additional file 9. Data management and server maintenance user checklist, EN-BIRTH study. [file 12884_2020_3426_MOESM9_ESM.pdf]

SUPPLEMENT TITLE:
Every Newborn BIRTH multi-country validation study: informing measurement of coverage and quality of maternal and newborn care

PAPER TITLE:
Electronic data collection for multi-country, hospital-based, clinical observation of maternal and newborn care: EN-BIRTH study experiences

Additional file 9: Data management and server maintenance user checklist, EN-BIRTH study

This user checklist aims to support data managers' adherence to EN-BIRTH data management operating procedures across all EN-BRITH study sites. Use of this check-list aims to reduce risks of data loss or data security breach.

**SERVER MAINTENANCE**	**Frequency**	**Completed**
Keep OS software update is ON	daily	
Set Antivirus software update hourly	daily	
Check server for malware and viruses	monthly	
Reset server password	monthly	
Review server's disk, CPU, RAM and network utilization	weekly	
Check remaining server storage space	daily	
Check remaining [backup 1] storage space	weekly	
Check remaining [backup 2] storage space	weekly	
Update Hosting or Server Control Panel	monthly	
Run hardware diagnostics and logs for any errors (overheating, disk read error, network failure)	monthly	
**SERVER BACKUP**		
Back up ENAP SQL Database [1st copy] at 07:00 PM on a different (local) drive	daily	
Back up 1st copy to (remote) external drive [2nd copy] after 1st copy is created	daily	
Compress and encrypt 2nd back up copy and store in a secure (off-site) location	daily	
Stored passwords for BACKUP files on paper (2 copies) in a secure location and in PGP encrypted digital file	daily	
Verify backup files are working	monthly	
Delete old logs, after verification of backup files	monthly	
**HARDWARE-tablets**		
Tablet ANDROID OS software is up-to-date check	monthly	
ENAP app is up-to-date	weekly	
